# Association between adjuvant therapy and survival in colorectal cancer patients according to metabolic Warburg-subtypes

**DOI:** 10.1007/s00432-023-04581-w

**Published:** 2023-02-01

**Authors:** Kelly Offermans, Josien C. A. Jenniskens, Colinda C. J. M. Simons, Iryna Samarska, Gregorio E. Fazzi, Kim M. Smits, Leo J. Schouten, Matty P. Weijenberg, Heike I. Grabsch, Piet A. van den Brandt

**Affiliations:** 1 Department of Epidemiology, GROW School for Oncology and Reproduction, Maastricht University Medical Center+, Maastricht, The Netherlands; 2 Department of Pathology, GROW School for Oncology and Reproduction, Maastricht University Medical Center+, Maastricht, The Netherlands; 3 Pathology and Data Analytics, Leeds Institute of Medical Research at St James’s, University of Leeds, Leeds, UK; 4 Department of Epidemiology, Care and Public Health Research Institute (CAPHRI), Maastricht University Medical Center+, Maastricht, The Netherlands

**Keywords:** Warburg-effect, Colorectal cancer, Survival, Chemotherapy, Radiotherapy, Adjuvant therapy

## Abstract

**Purpose:**

Tumor location and tumor node metastasis (TNM) stage guide treatment decisions in colorectal cancer (CRC) patients. However, patients with the same disease stage do not benefit equally from adjuvant therapy. Hence, there remains an urgent clinical need to identify prognostic and/or predictive biomarker(s) to personalize treatment decisions. In this exploratory study, we investigated whether our previously defined metabolic Warburg-subtypes can predict which CRC patients might derive survival benefit from adjuvant therapy.

**Methods:**

Information regarding treatment (surgery only: *n* = 1451; adjuvant radiotherapy: *n* = 82; or adjuvant chemotherapy: *n* = 260) and Warburg-subtype (Warburg-low: *n* = 485, -moderate: *n* = 641, or –high: *n* = 667) was available for 1793 CRC patients from the Netherlands Cohort Study (NLCS). Kaplan–Meier curves and Cox regression models were used to investigate survival benefit from adjuvant therapy compared to surgery-only for the different Warburg-subtypes.

**Results:**

Patients with Warburg-moderate CRC (HR_CRC-specific_ 0.64; 95% CI 0.47–0.86, HR_overall_ 0.61; 95% CI 0.47–0.80), and possibly Warburg-high CRC (HR_CRC-specific_ 0.86; 95% CI 0.65–1.14, HR_overall_ 0.82; 95% CI 0.64–1.05), had survival benefit from adjuvant therapy. No survival benefit was observed for patients with Warburg-low CRC (HR_CRC-specific_ 1.07; 95% CI 0.76–1.52, HR_overall_ 0.95; 95% CI 0.70–1.30). There was a significant interaction between Warburg-subtype and adjuvant therapy for CRC-specific survival (*p* = 0.049) and overall survival (*p* = 0.035).

**Conclusion:**

Our results suggest that Warburg-subtypes may predict survival benefit from adjuvant therapy in CRC patients. A survival benefit from adjuvant therapy was observed for patients with Warburg-moderate and possibly Warburg-high CRC, but not for patients with Warburg-low CRC. Future prospective studies are necessary to validate our findings.

**Supplementary Information:**

The online version contains supplementary material available at 10.1007/s00432-023-04581-w.

## Introduction

Colorectal cancer (CRC) is the third most commonly diagnosed cancer and the second-leading cause of cancer-related death worldwide, accounting for more than 900,000 deaths in 2020 (Rawla et al. 2019; Ferlay et al. 2020). Currently, tumor location and tumor node metastasis (TNM) stage guide treatment decisions in CRC patients (Kawakami et al. 2015; Roelands et al. 2017). However, patients with the same disease stage can have different survival and response to adjuvant therapy (Kawakami et al. 2015; Sinicrope et al. 2016, Roelands et al. 2017; Zhai et al. 2017; Ji et al. 2018). This may be due to heterogeneity in patient or tumor characteristics (Kawakami et al. 2015; Sinicrope et al. 2016; Roelands et al. 2017; Zhai et al. 2017; Ji et al. 2018).

Currently, there is only a limited number of biomarkers to identify CRC patients who are most likely to benefit from adjuvant therapy (Ji et al. 2018). Molecular classification of CRC may identify patient subgroups at high risk for recurrence and death, thereby facilitating the selection of patients for (personalized) therapy (Kawakami et al. 2015; Sinicrope et al. 2016). However, to date, only assessment of DNA mismatch repair (MMR) status and *RAS* and *BRAF* mutation status have been integrated into routine clinical practice to select patients for specific therapies (Fontana et al. 2019, Ten Hoorn et al. 2022). Hence, there remains an urgent clinical need to identify novel prognostic and/or predictive biomarker(s) to improve survival and quality of life in CRC patients (Ji et al. 2018, Ten Hoorn et al. 2022).

Metabolic reprogramming is one of the recognized hallmarks of cancer (Hanahan and Weinberg 2011). Otto Warburg first described in the 1920s, that cancer cells increase their glucose uptake and lactate secretion, even in the presence of oxygen (Warburg et al. 1927; Bensinger and Christofk 2012; Kato et al. 2018; Wolpaw and Dang 2018). This phenomenon of aerobic glycolysis, also known as the “Warburg-effect”, has since been observed in a variety of cancer types, including CRC (Sakashita et al. 2001; Potter et al. 2016).

We previously classified CRC as Warburg-low (i.e., low probability of the presence of the Warburg-effect), Warburg-moderate, or Warburg-high using a pathway-based sum score based on the expression levels of six glycolytic proteins, including transcriptional regulators, indicative of the Warburg-effect (LDHA, GLUT1, MCT4, PKM2, p53, and PTEN) (Jenniskens et al. 2021a; Offermans et al. 2021; Jenniskens et al. 2022). Our previous results, based on the total cohort of CRC patients, indicated that the Warburg-high subtype was associated with a poor survival in CRC patients, independent of known prognostic factors like TNM stage (Offermans et al. 2021).

Many studies have investigated the relationship between cellular metabolism and therapy resistance in CRC (Liu et al. 2021). The majority of studies suggested that the Warburg-effect promotes tumor characteristics that contribute to adjuvant therapy resistance (Morandi and Indraccolo 2017; Zhong and Zhou 2017; Zaal and Berkers 2018; Desbats et al. 2020; Kitazawa et al. 2020; Liu et al. 2021; Dong et al. 2022). However, most current evidence is based on in vitro cell culture studies, whereas—to the best of our knowledge—evidence from prospective cohort studies is lacking.

We hypothesized that patients with Warburg-high CRC will not derive a survival benefit from adjuvant chemo- or radiotherapy, whereas patients with Warburg-low CRC will derive survival benefit from adjuvant therapy. In this exploratory study, we therefore aimed to investigate whether our previously defined Warburg-subtypes can be used to predict survival benefit from adjuvant therapy in CRC patients.

## Methods

### Design and study population

The population-based series of colorectal cancer (CRC) patients in this study was derived from the prospective Netherlands Cohort Study (NLCS), which has been described in detail previously (van den Brandt et al. 1990a). Briefly, the NLCS was initiated in September 1986 and included 120,852 men and women, aged 55–69 years old, who completed a mailed, self-administered questionnaire on diet and other cancer risk factors at baseline (van den Brandt et al. 1990a). Participants agreed to participate in the study by completing and returning the questionnaire.

**Fig. 1:**
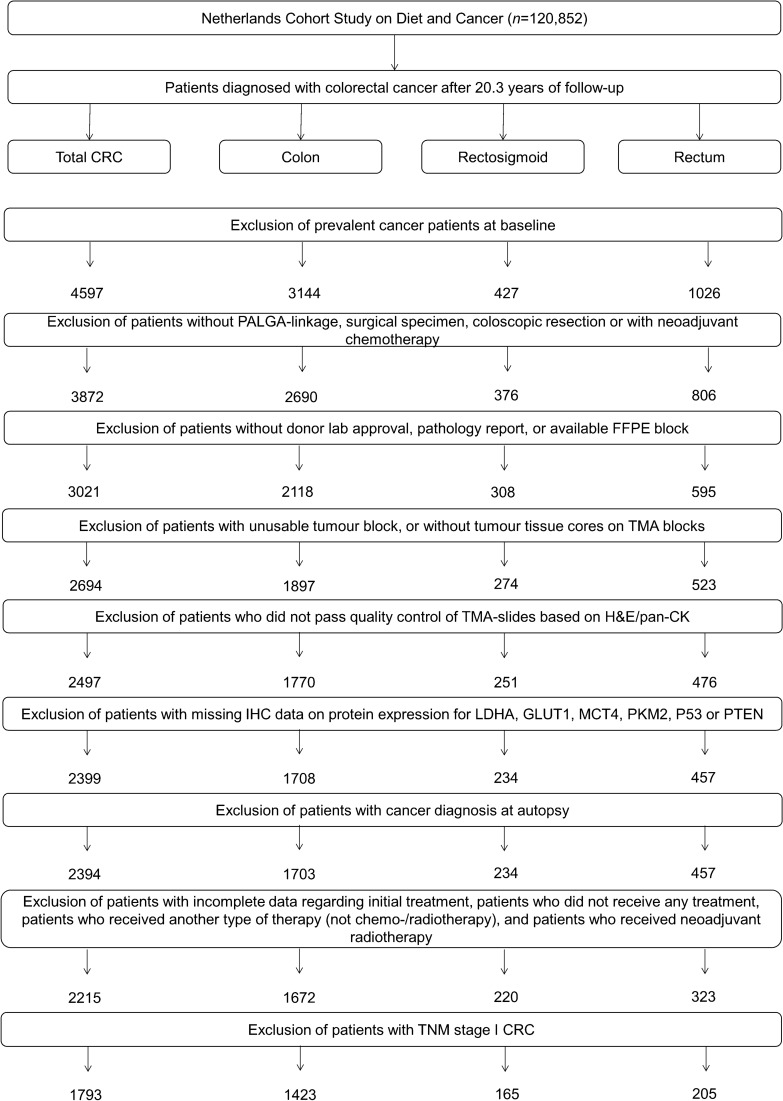
Flow diagram of the number of CRC patients available for analyses in the Netherlands Cohort Study (NLCS), 1986–2006. *CRC* colorectal cancer, *PALGA* Netherlands pathology database, *TMA* Tissue MicroArray

The entire prospective cohort was followed up for cancer incidence by annual record linkage with the Netherlands Cancer Registry and PALGA, the nationwide Dutch Pathology Registry (van den Brandt et al. 1990b; Casparie et al. 2007), covering 20.3 years of follow-up (September 17, 1986 until January 1, 2007). The completeness of cancer incidence follow-up was estimated to be > 96% (Goldbohm et al. 1994). After excluding patients who reported a history of cancer (excluding non-melanoma skin cancer) at baseline, 4597 incident CRC patients were available (Fig. 1).

The NLCS was approved by the institutional review boards of the TNO Quality of Life Research Institute (Zeist, the Netherlands) and Maastricht University (Maastricht, the Netherlands). Ethical approval for this study was obtained from the Medical Ethical Committee (METC) of Maastricht University Medical Center + .

### Establishing Warburg-subtypes based on Immunohistochemistry

Formalin-fixed paraffin-embedded (FFPE) tissue blocks from CRC resection specimens, excluding CRC patients who received neo-adjuvant chemotherapy (*n* = 10), were collected as part of the Rainbow-Tissue MicroArray (TMA) project (van den Brandt 2018). Details regarding TMA construction have been described previously (Offermans et al. 2021).

In total, 78 TMA blocks were constructed containing three 0.6 mm cores from tumor and three from normal epithelium of 2694 CRC patients (Fig. 1). Serial sections (5 µm) were subjected to immunohistochemistry (IHC) for Warburg-related proteins (LDHA, GLUT1, MCT4, PKM2, p53, PTEN) and mismatch-repair (MMR)-related proteins (MLH1, MSH2), as described previously (Jenniskens et al. 2021a, b; Offermans et al. 2021; Jenniskens et al. 2022).

Requiring at least one tumor core per patient, 2497 CRC patients passed quality control (Fig. 1). Multiple core-level IHC scores were combined into patient-level Warburg-subtypes as described previously (Jenniskens et al. 2021a, b; Offermans et al. 2021; Jenniskens et al. 2022). After excluding patients with missing IHC data, 2394 CRC patients were categorized as “Warburg-low” (*n* = 695, 29.0%), “Warburg-moderate” (*n* = 858, 35.8%) or “Warburg-high” (*n* = 841, 35.1%) subtype.

### Clinical characteristics and follow-up

Follow-up for vital status of the CRC patients was carried out through linkage to the Central Bureau of Genealogy and the municipal population registries until December 31, 2012. Patients who were found to have CRC at autopsy (*n* = 5), patients with incomplete data regarding initial treatment (*n* = 21), patients who did not receive any treatment (no surgery, chemo- or radiotherapy; *n* = 8), patients who received another type of therapy (*n* = 7), or patients who received neo-adjuvant radiotherapy (*n* = 143) were excluded. Furthermore, patients with TNM stage I CRC (*n* = 422), who were mostly treated with surgery only (*n* = 412, 97.6%), were excluded from analyses to ensure that patients in the surgery only subgroup had similar clinical characteristics as patients in the adjuvant therapy subgroup. Hence, 1,793 CRC patients were available for analyses (Fig. 1).

Causes of death were retrieved from Statistics Netherlands. CRC-specific deaths included patients with an underlying cause attributed to malignant neoplasms of the colon, rectosigmoid junction, or rectum. Overall vital status was available for 1,792 (99.9%) patients and CRC-specific vital status for 1,765 (98.4%) patients.

Information about age at diagnosis, pTNM stage, tumor location, tumor differentiation grade, and primary adjuvant therapy (i.e., treatments included in the initial treatment plan drawn up after diagnosis) was retrieved from the cancer registry or PALGA histopathology reports. The cancer registry only registered information regarding the primary treatment that was performed.

### Statistical analyses

Descriptive statistics were calculated for clinical characteristics, using mean (standard deviation) or median (range) for continuous data and frequencies (percentage) for categorical data. For categorical variables, differences across treatment subgroups (i.e., surgery only, surgery and adjuvant radiotherapy, surgery and adjuvant chemotherapy) were evaluated using chi-squared (*χ*^2^) tests. For continuous variables, the distributions across groups were evaluated using Kruskal–Wallis tests.

The primary outcomes were CRC-specific survival (time from CRC diagnosis to CRC-related death or end of follow-up) and overall survival (time from CRC diagnosis to death from any cause or end of follow-up). Survival analyses were restricted to 10 years of follow-up because of the limited number of events in the later period (CRC-specific deaths: *n* = 22; overall deaths: *n* = 175). Kaplan–Meier curves were estimated to examine survival benefit from adjuvant therapy for the different Warburg-subtypes (Warburg-low, Warburg-moderate, and Warburg-high). Differences between survival curves were investigated using Wilcoxon tests.

In addition, Cox proportional hazards regression was used to estimate Hazard ratios (HRs) and 95% confidence intervals (CIs) for associations between adjuvant therapy and survival by Warburg-subtype. The proportional hazards assumption was tested using the scaled Schoenfeld residuals (Schoenfeld 1982), by evaluating -log–log transformed survival curves or by introducing time–covariate interactions into the models. HRs were adjusted for a set of a priori selected prognostic factors: age at diagnosis (years); sex (men, women); tumor location (colon, rectosigmoid, rectum); pTNM stage (II, III, IV, unknown); differentiation grade (well, moderate, poor/undifferentiated, unknown); and MMR deficiency (no, yes, unknown). Year of diagnosis and pTNM version were considered as potential confounders and were retained in the models if they altered HRs by more than 10% (Kamangar 2012; Alexander et al. 2015). A separate category (‘unknown’) was used for patients with unknown clinical information regarding pTNM stage or differentiation grade to enable inclusion of these patients in the Cox proportional hazards models.

Disease stage was based on the pTNM classification according to the edition valid at the time of surgery, resulting in the use of five different TNM editions (UICC TNM editions 3–6), as described previously (Offermans et al. 2021). However, the main TNM stage groupings (I/II/III/IV) remained essentially unchanged (Sobin et al. 2010).

Sensitivity analyses, excluding CRC patients with unknown clinical information regarding TNM stage and differentiation grade (*n* = 143), yielded similar results (*data not shown*).

All analyses were conducted in Stata Statistical Software: Release 16 (StataCorp., College Station, TX). Two-sided *p* values < 0.05 were considered significant.

## Results

### Clinical characteristics

Clinical characteristics of the 1793 included colorectal cancer (CRC) patients according to therapy are presented in Table 1. The large majority (*n* = 1451, 80.9%) of CRC patients from the prospective Netherlands Cohort Study (NLCS) were treated with surgery only, while 82 (4.6%) and 260 (14.5%) patients were treated with adjuvant radio- or chemotherapy, respectively. The use of adjuvant chemotherapy increased over time (from 1.3% in 1986–1988 to 13.4% in 2004–2006), whereas the administration of adjuvant radiotherapy decreased (from 10.5% in 1986–1988 to 0.0% in 2004–2006; *p* < 0.001).

**Table 1:** Clinical characteristics of colorectal cancer patients (*n* = 1793) within the Netherlands Cohort Study (NLCS, 1986–2006), according to adjuvant therapy (surgery, surgery and radiotherapy, surgery and chemotherapy)

Clinical characteristics	Total CRC (*n* = 1793)	Surgery only (*n* = 1451)	Adjuvant therapy		*P* value^a^
			Surgery + RT (*n* = 82)	Surgery + CHT (*n* = 260)	
Year of diagnosis, *n* (%)					
1986–1988	76 (4.2)	67 (88.2)	8 (10.5)	1 (1.3)	< 0.001
1989–1991	149 (8.3)	118 (79.2)	18 (12.1)	13 (8.7)	
1992–1994	243 (13.6)	190 (78.2)	19 (7.8)	34 (14.0)	
1995–1997	336 (18.7)	262 (78.0)	20 (6.0)	54 (16.1)	
1998–2000	330 (18.4)	256 (77.6)	16 (4.9)	58 (17.6)	
2001–2003	323 (18.0)	267 (82.7)	1 (0.3)	55 (17.0)	
2004–2006	336 (18.7)	291 (86.6)	–	45 (13.4)	
Age at diagnosis in years, median (range)	74.0 (55.0–89.0)	75.0 (55.0–89.0)	69.0 (56.0–79.0)	72.0 (60.0–86.0)	< 0.001^b^
Sex, *n* (%)					
Men	980 (54.7)	766 (78.2)	53 (5.4)	161 (16.4)	0.004
Women	813 (45.3)	685 (84.3)	29 (3.6)	99 (12.2)	
Tumor location, *n* (%)					
Colon	1423 (79.4)	1201 (84.4)	10 (0.7)	212 (14.9)	< 0.001
Rectosigmoid	165 (9.2)	125 (75.8)	13 (7.9)	27 (16.4)	
Rectum	205 (11.4)	125 (61.0)	59 (28.8)	21 (10.2)	
pTNM stage, *n* (%)					
II	860 (48.0)	806 (93.7)	35 (4.1)	19 (2.2)	< 0.001
III	578 (32.2)	379 (65.6)	41 (7.1)	158 (27.3)	
IV	322 (18.0)	236 (73.3)	3 (0.9)	83 (25.8)	
Unknown	33 (1.8)	30 (90.9)	3 (9.1)	–	
Tumor extension (pT), *n* (%)					
T1	8 (0.5)	5 (62.5)	1 (12.5)	2 (25.0)	< 0.001
T2	69 (3.9)	41 (59.4)	7 (10.1)	21 (30.4)	
T3	1448 (80.6)	1188 (82.0)	62 (4.3)	198 (13.7)	
T4	229 (12.8)	182 (79.5)	9 (3.9)	38 (16.6)	
Unknown	39 (2.2)	35 (89.7)	3 (7.7)	1 (2.6)	
Lymph node involvement (pN), *n* (%)					
N0	817 (45.6)	752 (92.0)	32 (3.9)	33 (4.0)	< 0.001
N +	813 (45.3)	546 (67.2)	44 (5.4)	223 (27.4)	
Unknown	163 (9.1)	153 (93.9)	6 (3.7)	4 (2.5)	
Differentiation grade, *n* (%)					
Well	133 (7.4)	112 (84.2)	3 (2.3)	18 (13.5)	0.100
Moderate	1165 (65.0)	943 (80.9)	62 (5.3)	160 (13.7)	
Poor/undifferentiated	267 (20.5)	286 (77.9)	14 (3.8)	67 (18.3)	
Unknown	128 (7.2)	110 (85.9)	3 (2.3)	15 (11.7)	
dMMR, *n* (%)					
No	1560 (87.0)	1241 (79.6)	80 (5.1)	239 (15.3)	0.002
Yes	214 (11.9)	192 (89.7)	2 (0.9)	20 (9.4)	
Unknown	19 (1.1)	18 (94.7)	–	1 (5.3)	
Warburg-subtype, *n* (%)					
Warburg-low	485 (27.1)	395 (81.4)	23 (4.7)	67 (13.8)	0.950
Warburg-moderate	641 (35.8)	518 (80.8)	31 (4.8)	92 (14.4)	
Warburg-high	667 (37.2)	538 (80.7)	28 (4.2)	101 (15.1)	

*CRC* colorectal cancer, *RT* radiotherapy, *CHT* chemotherapy, *TNM* tumor node metastasis, *dMMR* mismatch repair-deficient

^a^*P* value for the *χ*^2^ test, unless otherwise specified

^b^*P* value for the Kruskal–Wallis test

CRC patients treated with adjuvant radio- or chemotherapy were younger compared to patients treated with surgery only (median age at diagnosis 69.0 years and 72.0 years versus 75.0 years, respectively; *p* < 0.001). Men were more frequently treated with adjuvant radio- or chemotherapy compared to women (5.4% and 16.4% of men versus 3.6% and 12.2% of women, respectively; *p* = 0.004). Patients with colon cancers were more often treated with surgery only compared to patients with rectosigmoid or rectal cancers (84.4% versus 75.8% and 61.0%, respectively; *p* < 0.001). Furthermore, patients with rectal cancers were more often treated with adjuvant radiotherapy compared to patients with rectosigmoid or colon cancers (28.8% versus 7.9% and 0.7%, respectively). Patients with pTNM stage III or IV CRC more often received adjuvant chemotherapy compared to patients with pTNM stage II CRC (27.3% and 25.8% versus 2.2%, respectively; *p* < 0.001). Patients who were treated with adjuvant radio- or chemotherapy were, in retrospect, more likely to have MMR proficient CRC (MMR_proficient_ 5.1% and 15.3% versus MMR_deficient_ 0.9% and 9.4%, respectively; *p* = 0.002).

### Warburg-subtypes and survival after adjuvant therapy

The median follow-up time since diagnosis was 3.72 years (range: 0.0027 to 25.49 years). Survival analyses were restricted to 10 years of follow-up, because of the limited number of events in the later period. During these first 10 years of follow-up, 1243 (69.3%) deaths were observed, of which 848 (68.2%) were CRC-related deaths.

#### Association between adjuvant therapy and survival according to Warburg-subtype

**Fig. 2:**
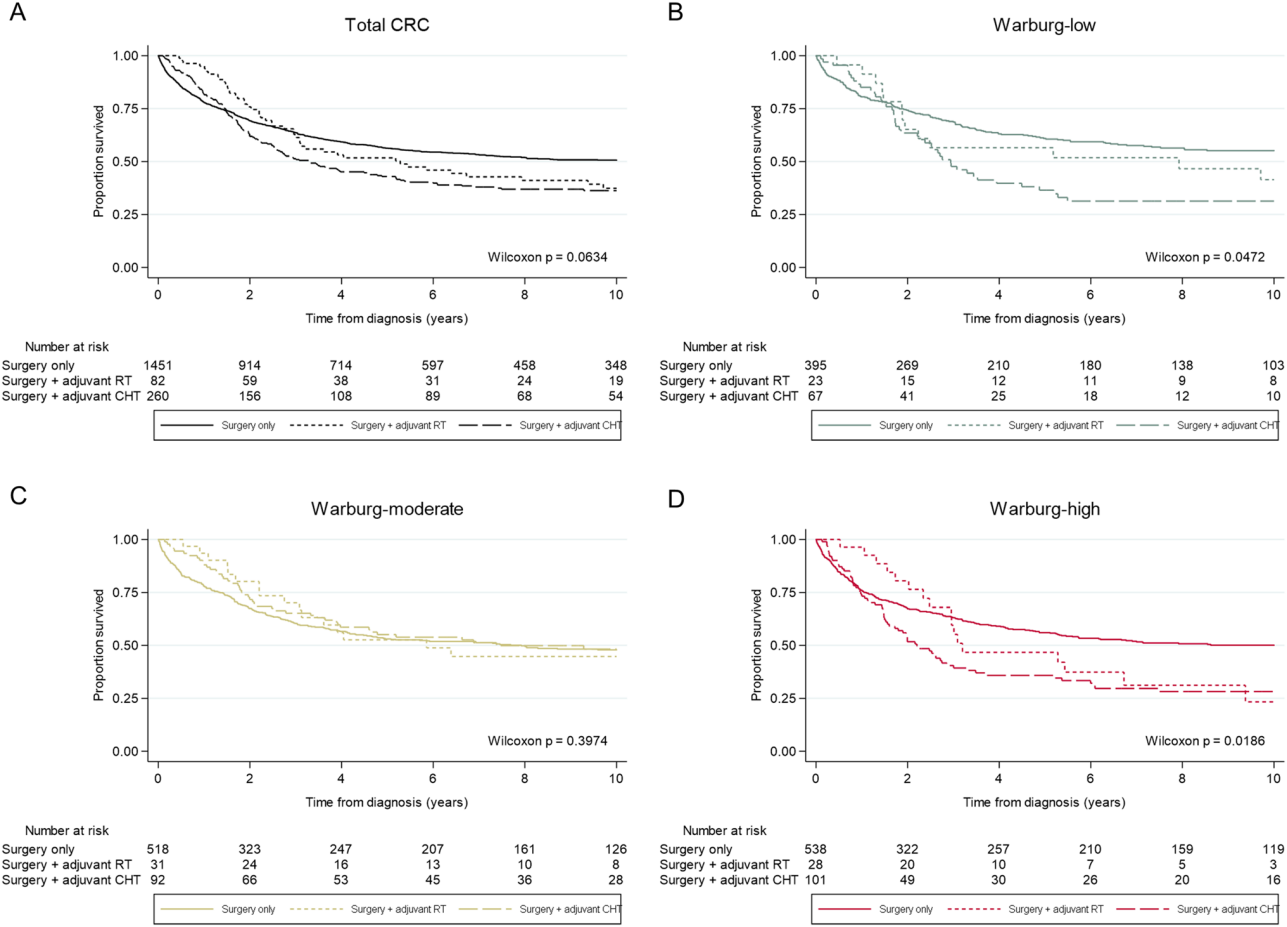
Univariable Kaplan–Meier curves showing CRC-specific survival of colorectal cancer patients within the Netherlands Cohort Study (NLCS, 1986–2006) for **A** Total CRC, **B** Warburg-low CRC, **C** Warburg-moderate CRC, or **D** Warburg-high CRC, according to the treatment received (surgery only, surgery and adjuvant radiotherapy, surgery adjuvant chemotherapy). *RT* radiotherapy, *CHT* chemotherapy

**Fig. 3:**
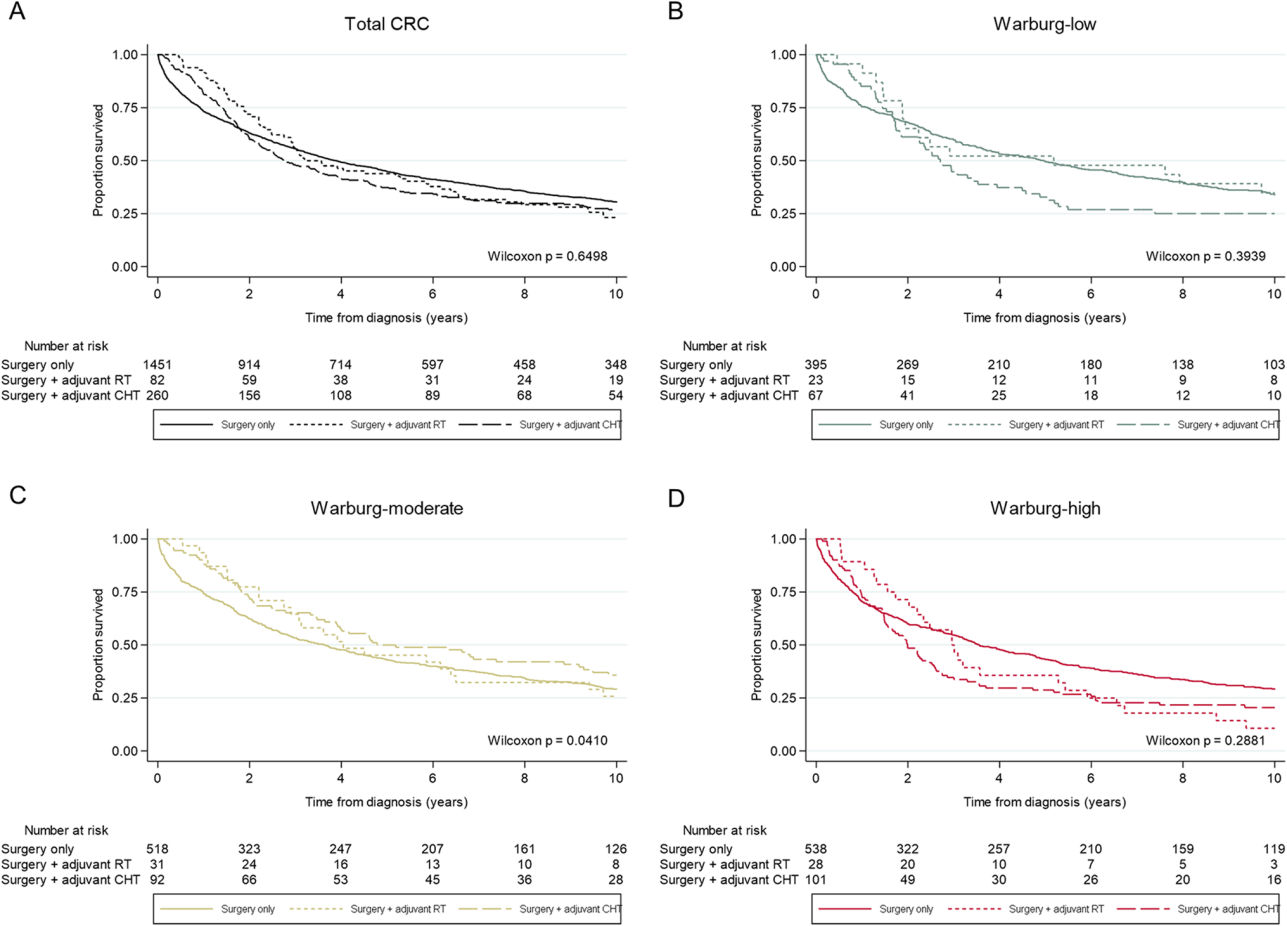
Univariable Kaplan–Meier curves showing overall survival of colorectal cancer patients within the Netherlands Cohort Study (NLCS, 1986–2006) for **A** Total CRC, **B** Warburg-low CRC, **C** Warburg-moderate CRC, or **D** Warburg-high CRC, according to the treatment received (surgery only, surgery and adjuvant radiotherapy, surgery and adjuvant chemotherapy). *RT* radiotherapy, *CHT* chemotherapy

In patients with Warburg-low CRC, univariable Kaplan–Meier curves showed significant differences in CRC-specific survival (p_CRC-specific_ = 0.047), but not overall survival (p_overall_ = 0.394), between treatment groups (Figs. 2A, 3A). Patients with Warburg-low CRC treated with adjuvant (chemo)therapy had a significantly worse CRC-specific survival compared to patients with Warburg-low CRC treated with surgery only (HR_adjuvant therapy_ 1.63; 95% CI 1.20–2.20 and HR_adjuvant chemotherapy_ 1.75; 95% CI 1.25–2.45; Table 2). These associations with survival disappeared after adjustment for confounders in multivariable-adjusted analyses (HR_adjuvant therapy_ 1.07; 95% CI 0.76–1.52 and HR_adjuvant chemotherapy_ 1.03; 95% CI 0.70–1.51; Table 2).

**Table 2:** Univariable and multivariable-adjusted hazard ratios for associations between adjuvant therapy (surgery, surgery plus radiotherapy, surgery plus chemotherapy) and CRC-specific and overall survival for the Warburg-subtypes (Warburg-low, Warburg-moderate, and Warburg-high) within the Netherlands Cohort Study (NLCS, 1986–2006)

	*N*	CRC-specific survival			Overall survival		
		CRC deaths (%)	HR (95% CI)		Deaths (%)	HR (95% CI)	
			Univariable	Multivariable-adjusted^a^		Univariable	Multivariable-adjusted^a^
Total CRC							
Surgery only	1451	644 (44.4)	1.00 (ref)	1.00 (ref)	992 (68.4)	1.00 (ref)	1.00 (ref)
Surgery + adjuvant therapy	342	204 (59.6)	1.31 (1.12–1.53)	0.80 (0.68–0.96)	251 (73.4)	1.07 (0.94–1.23)	0.78 (0.67–0.91)
Surgery + adjuvant RT	82	46 (56.1)	1.15 (0.85–1.55)	1.14 (0.81–1.61)	63 (76.8)	1.05 (0.81–1.35)	1.22 (0.91–1.63)
Surgery + adjuvant CHT	260	158 (60.8)	1.37 (1.15–1.63)	0.74 (0.61–0.90)	188 (72.3)	1.08 (0.93–1.27)	0.69 (0.58–0.82)
Warburg-low							
Surgery only	395	159 (40.3)	1.00 (ref)	1.00 (ref)	255 (64.6)	1.00 (ref)	1.00 (ref)
Surgery + adjuvant therapy	90	57 (63.3)	1.63 (1.20–2.20)	1.07 (0.76–1.52)	65 (72.2)	1.20 (0.91–1.57)	0.95 (0.70–1.30)
Surgery + adjuvant RT	23	13 (56.5)	1.31 (0.74–2.30)	1.26 (0.67–2.36)	15 (65.2)	0.93 (0.55–1.57)	1.17 (0.66–2.09)
Surgery + adjuvant CHT	67	44 (65.7)	1.75 (1.25–2.45)	1.03 (0.70–1.51)	50 (74.6)	1.31 (0.97–1.78)	0.90 (0.63–1.27)
Warburg-moderate							
Surgery only	518	245 (47.3)	1.00 (ref)	1.00 (ref)	362 (69.9)	1.00 (ref)	1.00 (ref)
Surgery + adjuvant therapy	123	62 (50.4)	0.92 (0.69–1.21)	0.64 (0.47–0.86)	81 (65.9)	0.81 (0.64–1.03)	0.61 (0.47–0.80)
Surgery + adjuvant RT	31	16 (51.6)	0.94 (0.57–1.56)	1.23 (0.67–2.24)	23 (74.2)	0.93 (0.61–1.42)	1.32 (0.80–2.18)
Surgery + adjuvant CHT	92	46 (50.0)	0.91 (0.66–1.25)	0.53 (0.38–0.75)	58 (63.0)	0.77 (0.58–1.02)	0.50 (0.37–0.67)
Warburg-high							
Surgery only	538	240 (44.6)	1.00 (ref)	1.00 (ref)	375 (69.7)	1.00 (ref)	1.00 (ref)
Surgery + adjuvant therapy	129	85 (65.9)	1.58 (1.23–2.02)	0.86 (0.65–1.14)	105 (81.4)	1.31 (1.05–1.62)	0.82 (0.64–1.05)
Surgery + adjuvant RT	28	17 (60.7)	1.29 (0.79–2.12)	1.22 (0.67–2.21)	25 (89.3)	1.29 (0.86–1.93)	1.25 (0.77–2.03)
Surgery + adjuvant CHT	101	68 (67.3)	1.67 (1.27–2.18)	0.81 (0.60–1.09)	80 (79.2)	1.31 (1.03–1.67)	0.75 (0.57–0.98)

*CRC* colorectal cancer, *HR* hazard ratio, *CI* confidence interval, *RT* radiotherapy, *CHT* chemotherapy

^a^Adjusted for age at diagnosis (years), sex (male/female), tumor location (colon/rectosigmoid/rectum), TNM stage (II, III, IV, unknown), differentiation grade (well/moderate/poor/undifferentiated/unknown), MMR deficiency (no/yes/unknown), and year of diagnosis (per 3 years)

In patients with Warburg-moderate CRC, univariable Kaplan–Meier curves showed significant differences in overall survival (p_overall_ = 0.041), but not CRC-specific survival (p_CRC-specific_ = 0.397), between treatment groups (Figs. 2B, 3B). Patients with Warburg-moderate CRC treated with adjuvant (chemo)therapy had a better overall survival compared to patients with Warburg-moderate CRC treated with surgery only (HR_adjuvant therapy_ 0.81; 95% CI 0.64–1.03 and HR_adjuvant chemotherapy_ 0.77; 95% CI 0.58–1.02; Table 2). In multivariable-adjusted analyses, these inverse associations with survival became even stronger and reached statistical significance for both CRC-specific (HR_adjuvant therapy_ 0.64; 95% CI 0.47–0.86 and HR_adjuvant chemotherapy_ 0.53; 95% CI 0.38–0.75; Table 2) and overall survival (HR_adjuvant therapy_ 0.61; 95% CI 0.47–0.80 and HR_adjuvant chemotherapy_ 0.50; 95% CI 0.37–0.67; Table 2).

In patients with Warburg-high CRC, univariable Kaplan–Meier curves showed significant differences in CRC-specific survival (p_CRC-specific_ = 0.019), but not overall survival (p_overall_ = 0.288), between treatment groups (Figs. 2B, 3B). Patients with Warburg-high CRC treated with adjuvant (chemo)therapy had a significantly worse CRC-specific (HR_adjuvant therapy_ 1.58; 95% CI 1.23–2.02, HR_adjuvant chemotherapy_ 1.67; 95% CI 1.27–2.18) and overall survival (HR_adjuvant therapy_ 1.31; 95% CI 1.05–1.62, HR_adjuvant chemotherapy_ 1.31; 95% CI 1.03–1.67) compared to patients with Warburg-high CRC treated with surgery only (Table 2). In multivariable-adjusted analyses, these associations with survival changed direction but did not reach statistical significance (CRC-specific survival: HR_adjuvant therapy_ 0.86; 95% 0.65–1.14; overall survival: HR_adjuvant therapy_ 0.82; 95% CI 0.64–1.05; Table 2). However, the association between adjuvant chemotherapy and overall survival did reach statistical significance (HR_adjuvant chemotherapy_ 0.75; 95% CI 0.57–0.98; Table 2).

The interaction between Warburg-subtype and adjuvant therapy as calculated in a multivariable-adjusted Cox proportional hazard model, adjusted for age at diagnosis, sex, tumor location, TNM stage, differentiation grade, MMR status and year of diagnosis was statistically significant for CRC-specific survival (*p* = 0.049) and overall survival (*p* = 0.035).

In stratified analyses according to disease stage (Supplementary Table S1), similar trends were observed for patients with pTNM stage III CRC. However, in patients with pTNM stage II CRC, no significant association between adjuvant therapy and survival was observed for any of the Warburg-subtypes. In contrast, in patients with pTNM stage IV CRC, a significantly better survival was observed for patients with Warburg-low or Warburg-moderate CRC receiving adjuvant (chemo)therapy compared to patients who received surgery only. In stratified analyses according to tumor location (Supplementary Table S2), a significantly better survival was observed for patients with Warburg-moderate or Warburg-high cancers located in the colon who received adjuvant (chemo)therapy compared to patients who received surgery only. Furthermore, a significant survival benefit was observed for patients with Warburg-moderate cancers located in the rectum who received adjuvant (radio)therapy.

## Discussion

In this large, population-based series of colorectal cancer (CRC) patients, we investigated whether our previously defined immunohistochemistry (IHC)-based Warburg-subtypes can be used to predict survival benefit from adjuvant therapy. Our results indicate that Warburg-subtypes may predict treatment benefit in CRC patients. While in general patients with stage II–IV CRC who received adjuvant (chemo)therapy had a significantly favorable CRC-specific and overall survival compared to patients who received surgery only, this benefit was only observed in patients with Warburg-moderate CRC. Patients with Warburg-high CRC also seemed to benefit from adjuvant therapy, but associations did not reach statistical significance. In contrast, no benefit from adjuvant (chemo)therapy was found for patients with Warburg-low CRC.

Since the 1950s, 5-fluorouracil (5-FU)-based chemotherapy remains the main pharmacological treatment modality for patients with CRC (Van der Jeught et al. 2018). Although the administration of chemotherapy can improve the survival of cancer patients, chemotherapy resistance remains a major problem (Liu et al. 2021). In CRC, 5-FU-based chemotherapy remains ineffective in approximately 30% of patients (Kitazawa et al. 2020). Hence, there remains an urgent clinical need to identify novel prognostic and/or predictive biomarker(s) to improve survival and quality of life in CRC patients (Ji et al. 2018; Ten Hoorn et al. 2022).

To the best of our knowledge, we are the first to prospectively investigate whether Warburg-subtypes are associated with adjuvant (chemo)therapy resistance in a large population-based cohort of CRC patients. Nevertheless, many studies have investigated the relationship between cellular metabolism and therapy resistance in vitro (Liu et al. 2021). Moreover, one retrospective study has investigated the relation between expression patterns of proteins related to the Warburg-effect and response to therapy in patient tissue samples (Kitazawa et al. 2020). On the one hand, the majority of studies suggest that aerobic glycolysis promotes tumor characteristics that contribute to adjuvant therapy resistance (Morandi and Indraccolo 2017; Zhong and Zhou 2017; Zaal and Berkers 2018; Desbats et al. 2020; Kitazawa et al. 2020; Liu et al. 2021; Dong et al. 2022). On the other hand, there are studies that suggest that therapy resistance is accompanied by a metabolic shift from aerobic glycolysis toward oxidative phosphorylation (OXPHOS) (Denise et al. 2015; Vellinga et al. 2015; Taniguchi et al. 2016). Assuming that the Warburg-high subtype represents CRC that rely mainly on aerobic glycolysis to meet their metabolic demands, whereas the Warburg-low subtype represents a more oxidative metabolic phenotype (i.e., OXPHOS), our results are in contrast with those of the majority of previous studies which showed that aerobic glycolysis is associated with adjuvant therapy resistance (Liu et al. 2021; Dong et al. 2022; Zaal and Berkers 2018; Desbats et al. 2020; Zhong and Zhou 2017; Morandi and Indraccolo 2017).

Even though future studies are necessary to validate our results and to further investigate the biological mechanisms, the discrepancy in results might be explained by the fact that previous reports were mostly based on in vitro cell culture studies (Morandi and Indraccolo 2017) or were conducted retrospectively (Kitazawa et al. 2020). It has been reported that in vitro conditions differ drastically from the conditions found in vivo in the tumor microenvironment (Pampaloni et al. 2007; Vermeersch et al. 2014). Furthermore, it has been suggested that the effect of therapy might differ depending on the environment in which the cancer cells reside (Jo et al. 2018). For example, research suggests that cancer cells may be sensitive to chemotherapy in cell culture, but become resistant when transplanted into animal models (Trédan et al. 2007).

A potential explanation for the observation that patients with Warburg-low CRC had no survival benefit from adjuvant (chemo)therapy has been described by Vellinga et al*.* (2015)*.* Normally, the amount of adenosine 5’-triphosphate (ATP) that is generated by aerobic glycolysis is sufficient to support tumor cell growth and basal DNA repair activity (Gottesman et al. 2002; Vellinga et al. 2015). However, when chemotherapy is administered, the cellular ATP demand in cancer cells increases significantly as many enzymes involved in DNA repair, drug efflux, and drug detoxification require ATP to function (Gottesman et al. 2002; Vellinga et al. 2015). As OXPHOS is the most efficient way to generate ATP (Vander Heiden et al. 2009), cancer cells may switch from aerobic glycolysis to OXPHOS at times of high ATP demand (Vellinga et al. 2015). In line with our results, this may suggest that patients with Warburg-low CRC (i.e., patients with cancers that rely mainly on oxidative metabolism) are more capable of repairing DNA damage and regulating drug metabolism compared to patients with Warburg-moderate and Warburg-high CRC (i.e., patients with cancers that rely mainly on aerobic glycolysis), rendering them more resistant to adjuvant therapy.

Our results suggest that the predictive value of Warburg-subtypes may be limited to TNM stage III CRC. In TNM stage II, no survival benefit from adjuvant (chemo)therapy was observed for any of the Warburg-subtypes, while in TNM stage IV, all CRC patients had survival benefit from adjuvant (chemo)therapy regardless of Warburg-subtype. As adjuvant chemotherapy is the standard of care for TNM stage III CRC (Kornmann et al. 2008), and chemotherapy resistance is still a major problem in clinical practice (Kitazawa et al. 2020; Liu et al. 2021), Warburg-subtypes may in future help to determine which stage III CRC patients will benefit most from adjuvant (chemo)therapy.

The main strengths of the present study include the use of a large population-based series of incident CRC patients, the prospective design, the nearly complete follow-up, and the availability of tumor material for a large number of CRC patients. Our study has some limitations. First, we did not have a validation cohort available to confirm the observed associations. Second, we did not have any detailed clinical information available regarding the dosage, duration or exact type of treatment. Third, we did not adjust for multiple testing which may have potentially resulted in chance findings. Fourth, in the Netherlands Cohort Study (NLCS), the large majority of CRC patients were treated with surgery only, resulting in a relatively small number of patients that were treated with adjuvant therapy, thereby limiting the power of our analyses. However, the limited amount of patients treated with adjuvant therapy was representative for this time period (1986–2006) (Van Steenbergen et al. 2010). Lastly, limitations with regard to Warburg-subtyping were described in detail previously (Offermans et al. 2021).

## Conclusion

In conclusion, Warburg-subtypes may predict treatment benefit in CRC patients. Our results suggest that survival benefit from adjuvant (chemo)therapy in patients with CRC may depend on Warburg-subtype. Opposite to expectation, a survival benefit from adjuvant (chemo)therapy was observed for patients with Warburg-moderate and possibly also Warburg-high CRC, but not for patients with Warburg-low CRC.

All in all, our results highlight the importance of molecular classification of CRC based on Warburg-related proteins, in addition to TNM stage and tumor location, to identify subgroups of patients who are more likely to benefit from adjuvant (chemo)therapy. However, as this is an exploratory study, our results should be interpreted with caution and future prospective studies are necessary to validate our findings.

## Supplementary Information

Below is the link to the electronic supplementary material.

Supplementary file1 (DOCX 42 KB)

## Data Availability

The datasets generated and/or analysed during the current study are not publicly available because the informed consent does not allow for that.

## Abbreviations

5-FU: 5-Fluorouracil
ATP: Adenosine 5'-triphosphate
CI: Confidence interval
CRC: Colorectal cancer
FFPE: Formalin-fixed paraffin-embedded
HR: Hazard ratio
METC: Medical Ethical Committee
MMR: Mismatch repair
NLCS: Netherlands Cohort Study
OXPHOS: Oxidative phosphorylation
PALGA: Dutch Pathology Registry
TMA: Tissue MicroArray
TNM: Tumor node metastasis
