# Supplementary material for: Association between adjuvant therapy and survival in colorectal cancer patients according to metabolic Warburg-subtypes
Source: J Cancer Res Clin Oncol. 2023 Feb 1;149(9):6271–82. doi: 10.1007/s00432-023-04581-w (PMC10356897; doi:10.1007/s00432-023-04581-w)
Supplement: Supplementary file 1 — Supplementary file1 (DOCX 42 KB) [file 432_2023_4581_MOESM1_ESM.docx]

**Supplementary Table S1 –** Univariable and multivariable-adjusted hazard ratios for associations between adjuvant therapy (surgery, surgery plus radiotherapy, surgery plus chemotherapy) and CRC-specific and overall survival for the Warburg-subtypes (Warburg-low, Warburg-moderate, and Warburg-high) within the Netherlands Cohort Study (NLCS, 1986-2006), stratified on TNM stage (II/III/IV).

|  |  | | | | N |  | **CRC-specific survival** | | |  | **Overall survival** | | |
| --- | --- | --- | --- | --- | --- | --- | --- | --- | --- | --- | --- | --- | --- |
|  |  | | | |  |  | CRC deaths (%) | HR (95% CI) | |  | Deaths (%) | HR (95% CI) | |
|  |  | | | |  |  |  | Univariable | Multivariable-adjusted^a^ |  |  | Univariable | Multivariable-adjusted^a^ |
|  |  | | | |  |  |  |  |  |  |  |  |  |
| **TNM stage II** | | | | |  |  |  |  |  |  |  |  |  |
|  | **Warburg-low** | | | |  |  |  |  |  |  |  |  |  |
|  |  | Surgery only | | | 230 |  | 59 (25.7) | 1.00 (ref) | 1.00 (ref) |  | 126 (54.8) | 1.00 (ref) | 1.00 (ref) |
|  |  | Surgery + adjuvant therapy | | | 15 |  | 5 (33.3) | 1.28 (0.51-3.19) | 1.63 (0.60-4.45) |  | 8 (53.3) | 0.94 (0.46-1.93) | 1.39 (0.65-2.98) |
|  |  |  | *Surgery + adjuvant RT* | | *9* |  | *4 (44.4)* | *1.86 (0.67-5.12)* | *2.71 (0.77-9.47)* |  | *6 (66.7)* | *1.31 (0.58-2.98)* | *2.54 (0.98-6.56)* |
|  |  |  | *Surgery + adjuvant CHT* | | *6* |  | *1 (16.7)* | *0.57 (0.08-4.11)* | *0.72 (0.10-5.39)* |  | *2 (33.3)* | *0.51 (0.13-2.07)* | *0.63 (0.15-2.62)* |
|  | **Warburg-moderate** | | | |  |  |  |  |  |  |  |  |  |
|  |  | Surgery only | | | 278 |  | 80 (28.8) | 1.00 (ref) | 1.00 (ref) |  | 155 (55.8) | 1.00 (ref) | 1.00 (ref) |
|  |  | Surgery + adjuvant therapy | | | 19 |  | 6 (31.6) | 0.99 (0.43-2.26) | 1.39 (0.55-3.51) |  | 10 (52.6) | 0.85 (0.45-1.60) | 1.10 (0.54-2.23) |
|  |  |  | *Surgery + adjuvant RT* | | *14* |  | *6 (42.9)* | *1.43 (0.62-3.28)* | *1.91 (0.73-4.99)* |  | *9 (64.3)* | *1.14 (0.58-2.23)* | *1.57 (0.73-3.40)* |
|  |  |  | *Surgery + adjuvant CHT* | | *5* |  | *-* | *-* | *-* |  | *1 (20.0)* | *0.26 (0.04-1.83)* | *0.32 (0.04-2.39)* |
|  | **Warburg-high** | | | |  |  |  |  |  |  |  |  |  |
|  |  | Surgery only | | | 298 |  | 76 (25.5) | 1.00 (ref) | 1.00 (ref) |  | 167 (56.0) | 1.00 (ref) | 1.00 (ref) |
|  |  | Surgery + adjuvant therapy | | | 20 |  | 9 (45.0) | 1.91 (0.95-3.80) | 1.74 (0.76-3.96) |  | 14 (70.0) | 1.36 (0.79-2.34) | 1.49 (0.79-2.78) |
|  |  |  | *Surgery + adjuvant RT* | | *12* |  | *7 (58.3)* | *2.94 (1.35-6.41)* | *3.08 (1.16-8.21)* |  | *11 (91.7)* | *2.30 (1.25-4.24)* | *2.65 (1.25-5.61)* |
|  |  |  | *Surgery + adjuvant CHT* | | *8* |  | *2 (25.0)* | *0.85 (0.21-3.48)* | *0.75 (0.17-3.27)* |  | *3 (37.5)* | *0.54 (0.17-1.70)* | *0.63 (0.19-2.03)* |
|  |  |  | | |  |  |  |  |  |  |  |  |  |
| **TNM stage III** | | | | |  |  |  |  |  |  |  |  |  |
|  | **Warburg-low** | | | |  |  |  |  |  |  |  |  |  |
|  |  | Surgery only | | | 103 |  | 45 (43.7) | 1.00 (ref) | 1.00 (ref) |  | 69 (67.0) | 1.00 (ref) | 1.00 (ref) |
|  |  | Surgery + adjuvant therapy | | | 49 |  | 31 (63.3) | 1.33 (0.84-2.10) | 1.72 (0.95-3.11) |  | 34 (69.4) | 0.96 (0.64-1.45) | 1.07 (0.64-1.79) |
|  |  |  | | *Surgery + adjuvant RT* | *11* |  | *8 (72.7)* | *1.61 (0.76-3.41)* | *1.96 (0.76-5.05)* |  | *8 (72.3)* | *1.05 (0.50-2.18)* | *1.31 (0.54-3.17)* |
|  |  |  | | *Surgery + adjuvant CHT* | *38* |  | *23 (60.5)* | *1.25 (0.76-2.07)* | *1.67 (0.90-3.11)* |  | *26 (68.4)* | *0.94 (0.60-1.48)* | *1.02 (0.59-1.76)* |
|  | **Warburg-moderate** | | | |  |  |  |  |  |  |  |  |  |
|  |  | Surgery only | | | 137 |  | 74 (54.0) | 1.00 (ref) | 1.00 (ref) |  | 107 (78.1) | 1.00 (ref) | 1.00 (ref) |
|  |  | Surgery + adjuvant therapy | | | 77 |  | 31 (40.3) | 0.53 (0.35-0.80) | 0.46 (0.29-0.75) |  | 45 (58.4) | 0.51 (0.36-0.72) | 0.49 (0.33-0.73) |
|  |  |  | *Surgery + adjuvant RT* | | *15* |  | *8 (53.3)* | *0.82 (0.40-1.71)* | *1.01 (0.37-2.75)* |  | *12 (80.0)* | *0.85 (0.47-1.54)* | *1.36 (0.59-3.16)* |
|  |  |  | *Surgery + adjuvant CHT* | | *62* |  | *23 (37.1)* | *0.47 (0.29-0.75)* | *0.40 (0.23-0.67)* |  | *33 (53.2)* | *0.44 (0.30-0.65)* | *0.39 (0.25-0.62)* |
|  | **Warburg-high** | | | |  |  |  |  |  |  |  |  |  |
|  |  | Surgery only | | | 139 |  | 78 (56.1) | 1.00 (ref) | 1.00 (ref) |  | 111 (79.9) | 1.00 (ref) | 1.00 (ref) |
|  |  | Surgery + adjuvant therapy | | | 73 |  | 44 (60.3) | 0.87 (0.60-1.25) | 0.82 (0.54-1.25) |  | 55 (75.3) | 0.76 (0.55-1.05) | 0.80 (0.55-1.16) |
|  |  |  | *Surgery + adjuvant RT* | | *15* |  | *10 (66.7)* | *0.99 (0.51-1.92)* | *0.58 (0.24-1.39)* |  | *13 (86.7)* | *0.92 (0.52-1.63)* | *0.66 (0.31-1.39)* |
|  |  |  | *Surgery + adjuvant CHT* | | *58* |  | *34 (58.6)* | *0.84 (0.56-1.25)* | *0.90 (0.57-1.41)* |  | *42 (72.4)* | *0.72 (0.51-1.03)* | *0.85 (0.57-1.27)* |
|  |  |  | | |  |  |  |  |  |  |  |  |  |
| **TNM stage IV** | | | | |  |  |  |  |  |  |  |  |  |
|  | **Warburg**-**low** | | | |  |  |  |  |  |  |  |  |  |
|  |  | Surgery only | | | 55 |  | 51 (92.7) | 1.00 (ref) | 1.00 (ref) |  | 55 (100.0) | 1.00 (ref) | 1.00 (ref) |
|  |  | Surgery + adjuvant therapy | | | 24 |  | 21 (87.5) | 0.56 (0.34-0.94) | 0.39 (0.20-0.80) |  | 23 (95.8) | 0.56 (0.34-0.92) | 0.40 (0.21-0.79) |
|  |  |  | *Surgery + adjuvant RT* | | *1* |  | *1 (100.0)* | *0.55 (0.08-4.00)* | *0.50 (0.06-4.51)* |  | *1 (100.0)* | *0.51 (0.07-3.71)* | *0.37 (0.04-3.17)* |
|  |  |  | *Surgery + adjuvant CHT* | | *23* |  | *20 (87.0)* | *0.56 (0.33-0.95)* | *0.38 (0.18-0.82)* |  | *22 (95.7)* | *0.56 (0.34-0.93)* | *0.41 (0.20-0.85)* |
|  | **Warburg-moderate** | | | |  |  |  |  |  |  |  |  |  |
|  |  | Surgery only | | | 90 |  | 83 (92.2) | 1.00 (ref) | 1.00 (ref) |  | 90 (100.0) | 1.00 (ref) | 1.00 (ref) |
|  |  | Surgery + adjuvant therapy | | | 27 |  | 25 (92.6) | 0.58 (0.37-0.92) | 0.38 (0.21-0.68) |  | 26 (96.3) | 0.56 (0.36-0.87) | 0.36 (0.20-0.64) |
|  |  |  | *Surgery + adjuvant RT* | | *2* |  | *2 (100.0)* | *0.74 (0.18-3.01)* | *0.38 (0.03-4.19)* |  | *2 (100.0)* | *0.70 (0.17-2.86)* | *0.48 (0.05-4.57)* |
|  |  |  | *Surgery + adjuvant CHT* | | *25* |  | *23 (92.0)* | *0.57 (0.36-0.91)* | *0.38 (0.20-0.70)* |  | *24 (96.0)* | *0.55 (0.35-0.87)* | *0.36 (0.20-0.65)* |
|  | **Warburg-high** | | | |  |  |  |  |  |  |  |  |  |
|  |  | Surgery only | | | 91 |  | 82 (90.0) | 1.00 (ref) | 1.00 (ref) |  | 91 (100.0) | 1.00 (ref) | 1.00 (ref) |
|  |  | Surgery + adjuvant therapy | | | 35 |  | 32 (91.4) | 0.66 (0.44-1.01) | 0.69 (0.40-1.18) |  | 35 (100.0) | 0.65 (0.43-0.96) | 0.63 (0.38-1.05) |
|  |  |  | *Surgery + adjuvant RT* | | *-* |  | *-* | *-* | *-* |  | *-* | *-* | *-* |
|  |  |  | *Surgery + adjuvant CHT* | | *35* |  | *32 (91.4)* | *0.66 (0.44-1.01)* | *0.69 (0.40-1.18)* |  | *35 (100.0)* | *0.65 (0.43-0.96)* | *0.63 (0.38-1.05)* |

^a^Adjusted for age at diagnosis (years), sex (male/female), tumor location (colon/rectosigmoid/rectum), differentiation grade (well/moderate/poor/undifferentiated/unknown), MMR deficiency (no/yes), year of diagnosis (per 3 years)

**Supplementary Table S2 –** Univariable and multivariable-adjusted hazard ratios for associations between adjuvant therapy (surgery, surgery plus radiotherapy, surgery plus chemotherapy) and CRC-specific and overall survival for the Warburg-subtypes (Warburg-low, Warburg-moderate, and Warburg-high) within the Netherlands Cohort Study (NLCS, 1986-2006), stratified on tumor location (colon/rectosigmoid/rectum).

|  |  | | | N |  | **CRC-specific survival** | | |  | **Overall survival** | | |
| --- | --- | --- | --- | --- | --- | --- | --- | --- | --- | --- | --- | --- |
|  |  | | |  |  | CRC deaths (%) | HR (95% CI) | |  | Deaths (%) | HR (95% CI) | |
|  |  | | |  |  |  | Univariable | Multivariable-adjusted^a^ |  |  | Univariable | Multivariable-adjusted^a^ |
|  |  | | |  |  |  |  |  |  |  |  |  |
| **Colon** | | | |  |  |  |  |  |  |  |  |  |
|  | **Warburg-low** | | |  |  |  |  |  |  |  |  |  |
|  |  | Surgery only | | 319 |  | 130 (40.8) | 1.00 (ref) | 1.00 (ref) |  | 206 (64.6) | 1.00 (ref) | 1.00 (ref) |
|  |  | Surgery + adjuvant therapy | | 56 |  | 34 (60.7) | 1.54 (1.05-2.25) | 0.93 (0.60-1.45) |  | 41 (73.2) | 1.22 (0.87-1.71) | 0.88 (0.60-1.30) |
|  |  |  | *Surgery + adjuvant RT* | *4* |  | *2 (50.0)* | *0.98 (0.24-3.98)* | *0.73 (0.17-3.08)* |  | *3 (75.0)* | *0.95 (0.30-2.97)* | *0.82 (0.25-2.65)* |
|  |  |  | *Surgery + adjuvant CHT* | *52* |  | *32 (61.5)* | *1.59 (1.08-2.35)* | *0.95 (0.60-1.49)* |  | *38 (73.1)* | *1.25 (0.88-1.77)* | *0.89 (0.60-1.32)* |
|  | **Warburg-moderate** | | |  |  |  |  |  |  |  |  |  |
|  |  | Surgery only | | 429 |  | 203 (47.3) | 1.00 (ref) | 1.00 (ref) |  | 299 (69.7) | 1.00 (ref) | 1.00 (ref) |
|  |  | Surgery + adjuvant therapy | | 82 |  | 43 (52.4) | 0.96 (0.69-1.33) | 0.54 (0.37-0.78) |  | 55 (67.1) | 0.83 (0.62-1.10) | 0.52 (0.38-0.72) |
|  |  |  | *Surgery + adjuvant RT* | *4* |  | *3 (75.0)* | *1.55 (0.50-4.85)* | *3.57 (1.06-11.95)* |  | *4 (100.0)* | *1.49 (0.56-4.02)* | *2.65 (0.93-7.49)* |
|  |  |  | *Surgery + adjuvant CHT* | *78* |  | *40 (51.3)* | *0.93 (0.66-1.30)* | *0.49 (0.33-0.71)* |  | *51 (65.4)* | *0.80 (0.59-1.08)* | *0.47 (0.34-0.66)* |
|  | **Warburg-high** | | |  |  |  |  |  |  |  |  |  |
|  |  | Surgery only | | 453 |  | 199 (43.9) | 1.00 (ref) | 1.00 (ref) |  | 312 (68.9) | 1.00 (ref) | 1.00 (ref) |
|  |  | Surgery + adjuvant therapy | | 84 |  | 54 (64.3) | 1.58 (1.17-2.14) | 0.77 (0.55-1.08) |  | 65 (77.4) | 1.27 (0.97-1.66) | 0.73 (0.54-0.99) |
|  |  |  | *Surgery + adjuvant RT* | *2* |  | *1 (50.0)* | *0.79 (0.11-5.64)* | *0.88 (0.12-6.47)* |  | *1 (50.0)* | *0.49 (0.07-3.45)* | *0.51 (0.07-3.72)* |
|  |  |  | *Surgery + adjuvant CHT* | *82* |  | *53 (64.6)* | *1.61 (1.19-2.19)* | *0.77 (0.54-1.08)* |  | *64 (78.0)* | *1.30 (0.99-1.70)* | *0.74 (0.54-1.00)* |
|  |  |  | |  |  |  |  |  |  |  |  |  |
| **Rectosigmoid** | | | |  |  |  |  |  |  |  |  |  |
|  | **Warburg-low** | | |  |  |  |  |  |  |  |  |  |
|  |  | Surgery only | | 35 |  | 10 (28.6) | 1.00 (ref) | 1.00 (ref) |  | 25 (71.4) | 1.00 (ref) | 1.00 (ref) |
|  |  | Surgery + adjuvant therapy | | 13 |  | 8 (61.5) | 2.55 (1.00-6.50) | 2.98 (0.40-22.21) |  | 9 (69.2) | 1.20 (0.56-2.57) | 1.36 (0.31-6.05) |
|  |  |  | *Surgery + adjuvant RT* | *3* |  | *-* | *-* | *-* |  | *1 (33.3)* | *0.34 (0.05-2.52)* | *-* |
|  |  |  | *Surgery + adjuvant CHT* | *10* |  | *8 (80.0)* | *3.95 (1.53-10.19)* | *4.12 (0.53-31.87)* |  | *8 (80.0)* | *1.76 (0.78-3.94)* | *2.15 (0.45-10.20)* |
|  | **Warburg-moderate** | | |  |  |  |  |  |  |  |  |  |
|  |  | Surgery only | | 41 |  | 13 (31.7) | 1.00 (ref) | 1.00 (ref) |  | 26 (63.4) | 1.00 (ref) | 1.00 (ref) |
|  |  | Surgery + adjuvant therapy | | 15 |  | 7 (46.7) | 1.44 (0.57-3.62) | 0.71 (0.18-2.79) |  | 10 (66.7) | 1.08 (0.52-2.25) | 0.97 (0.38-2.46) |
|  |  |  | *Surgery + adjuvant RT* | *5* |  | *3 (60.0)* | *2.26 (0.64-8.02)* | *3.17 (0.46-22.00)* |  | *5 (100.0)* | *2.21 (0.83-5.86)* | *2.67 (0.73-9.82)* |
|  |  |  | *Surgery + adjuvant CHT* | *10* |  | *4 (40.0)* | *1.14 (0.37-3.48)* | *0.33 (0.07-1.65)* |  | *5 (50.0)* | *0.72 (0.28-1.88)* | *0.45 (0.13-1.56)* |
|  | **Warburg-high** | | |  |  |  |  |  |  |  |  |  |
|  |  | Surgery only | | 49 |  | 26 (53.1) | 1.00 (ref) | 1.00 (ref) |  | 38 (77.6) | 1.00 (ref) | 1.00 (ref) |
|  |  | Surgery + adjuvant therapy | | 12 |  | 9 (75.0) | 1.34 (0.62-2.89) | 1.00 (0.39-2.52) |  | 11 (91.7) | 1.21 (0.61-2.39) | 1.09 (0.48-2.45) |
|  |  |  | *Surgery + adjuvant RT* | *5* |  | *4 (80.0)* | *1.53 (0.53-4.42)* | *0.82 (0.15-4.32)* |  | *5 (100.0)* | *1.44 (0.56-3.72)* | *0.94 (0.22-4.01)* |
|  |  |  | *Surgery + adjuvant CHT* | *7* |  | *5 (71.4)* | *1.23 (0.47-3.21)* | *1.11 (0.34-3.54)* |  | *6 (85.7)* | *1.07 (0.45-2.54)* | *1.18 (0.43-3.25)* |
|  |  |  | |  |  |  |  |  |  |  |  |  |
| **Rectum** | | | |  |  |  |  |  |  |  |  |  |
|  | **Warburg**-**low** | | |  |  |  |  |  |  |  |  |  |
|  |  | Surgery only | | 41 |  | 19 (46.3) | 1.00 (ref) | 1.00 (ref) |  | 24 (58.5) | 1.00 (ref) | 1.00 (ref) |
|  |  | Surgery + adjuvant therapy | | 21 |  | 15 (71.4) | 1.55 (0.79-3.06) | 1.90 (0.54-6.63) |  | 15 (71.4) | 1.25 (0.65-2.38) | 1.89 (0.61-5.80) |
|  |  |  | *Surgery + adjuvant RT* | *16* |  | *11 (68.8)* | *1.58 (0.75-3.32)* | *2.86 (0.78-10.52)* |  | *11 (68.8)* | *1.25 (0.61-2.56)* | *2.60 (0.80-8.40)* |
|  |  |  | *Surgery + adjuvant CHT* | *5* |  | *4 (80.0)* | *1.49 (0.50-4.39)* | *0.52 (0.08-3.45)* |  | *4 (80.0)* | *1.24 (0.43-3.59)* | *0.79 (0.15-4.15)* |
|  | **Warburg-moderate** | | |  |  |  |  |  |  |  |  |  |
|  |  | Surgery only | | 48 |  | 29 (60.4) | 1.00 (ref) | 1.00 (ref) |  | 37 (77.1) | 1.00 (ref) | 1.00 (ref) |
|  |  | Surgery + adjuvant therapy | | 26 |  | 12 (46.2) | 0.62 (0.32-1.22) | 0.42 (0.14-1.25) |  | 16 (61.5) | 0.63 (0.35-1.14) | 0.33 (0.12-0.87) |
|  |  |  | *Surgery + adjuvant RT* | *22* |  | *10 (45.5)* | *0.62 (0.30-1.27)* | *0.45 (0.14-1.41)* |  | *14 (63.6)* | *0.66 (0.36-1.23)* | *0.37 (0.13-1.03)* |
|  |  |  | *Surgery + adjuvant CHT* | *4* |  | *2 (50.0)* | *0.63 (0.15-2.63)* | *0.34 (0.06-1.97)* |  | *2 (50.0)* | *0.47 (0.11-1.96)* | *0.21 (0.04-1.12)* |
|  | **Warburg-high** | | |  |  |  |  |  |  |  |  |  |
|  |  | Surgery only | | 36 |  | 15 (41.7) | 1.00 (ref) | 1.00 (ref) |  | 25 (69.4) | 1.00 (ref) | 1.00 (ref) |
|  |  | Surgery + adjuvant therapy | | 33 |  | 22 (66.7) | 1.78 (0.92-3.46) | 0.83 (0.34-2.07) |  | 29 (87.9) | 1.45 (0.84-2.48) | 1.13 (0.53-2.41) |
|  |  |  | *Surgery + adjuvant RT* | *21* |  | *12 (57.1)* | *1.36 (0.63-2.92)* | *0.72 (0.23-2.22)* |  | *19 (90.5)* | *1.34 (0.74-2.45)* | *1.42 (0.61-3.32)* |
|  |  |  | *Surgery + adjuvant CHT* | *12* |  | *10 (83.3)* | *2.88 (1.28-6.51)* | *1.00 (0.31-3.29)* |  | *10 (83.3)* | *1.69 (0.81-3.56)* | *0.73 (0.24-2.23)* |

^a^Adjusted for age at diagnosis (years), sex (male/female), TNM stage (II, III, IV, unknown), differentiation grade (well/moderate/poor/undifferentiated/unknown), MMR deficiency (no/yes), year of diagnosis (per 3 years)
